# DESIGN, MEASURES, AND CORRELATES OF CHANGES IN AD BLOOD BIOMARKERS AND ASSOCIATIONS WITH NEUROIMAGING OUTCOMES

**DOI:** 10.1093/geroni/igae098.0002

**Published:** 2024-12-31

**Authors:** Priya Palta, James Pike, Yifei Lu, Jinyu Chen, Keenan Walker, Kevin Sullivan, Thomas Mosley

**Affiliations:** University of North Carolina, Chapel Hill, North Carolina, United States; New York University, New York, New York, United States; Merck, Landsdale, Pennsylvania, United States; University of North Carolina, Chapel Hill, North Carolina, United States; National Institute on Aging, Baltimore, Maryland, United States; University of Mississippi Medical Center, Jackson, Mississippi, United States; University of Mississippi Medical Center - The MIND Center, Jackson, Mississippi, United States

## Abstract

Few studies have examined changes in blood-based biomarkers of AD pathology and neurodegeneration particularly during the mid- to late-life transition period and associations with brain pathology. Among 1525 (59.9% women, 25.8% Black) ARIC participants, we measured blood-based biomarkers of Aβ42, Aβ40, p-Tau181, NfL, and GFAP using stored specimens from midlife (1993-1995, mean age 58.3 years) and late-life (2011-2013, mean age 76.0 years followed to 2016-2019, mean age 80.7 years). Using linear mixed effects models, we examined whether known dementia-related risk factors in midlife (i.e., hypertension, diabetes, lipids, coronary heart disease, cigarette use, and physical activity) are associated with 20-year biomarker changes. We also examined the associations of midlife, late-life, and mid- to late-life changes in blood biomarkers with measures of whole brain cortical thickness, brain volume, and white matter hyperintensity volume (WMH) from a 3T brain MRI scan in multivariable linear regression models. All models were adjusted for age, sex, race-center, education, APOE, eGFR, body mass index, cardiovascular risk factors, and intracranial volume for volumetric analyses. Decreasing Aβ42/Aβ40 and increasing p-Tau181, NfL, and GFAP were observed from midlife to late-life. Midlife hypertension and diabetes were associated with a steeper rise in NfL and GFAP. Lower late-life levels of Aβ42/Aβ40 and higher late-life levels of p-Tau181, p-Tau181/Aβ42, NfL, and GFAP were associated with smaller cortical thickness and total brain volume, and larger WMH volume. Blood biomarkers of AD neuropathology, neuronal injury, and astrogliosis rise with age and are associated with known dementia risk factors and markers of neurodegeneration and cerebrovascular disease.

